# Antibiotic Prophylaxis and Postoperative Therapy in Tooth Extractions for Patients at Risk of Medication-Related Osteonecrosis of the Jaw (MRONJ): A Scoping Review

**DOI:** 10.3390/antibiotics14121279

**Published:** 2025-12-17

**Authors:** Selene Barone, Alessandro Antonelli, Antonio Madonna, Amerigo Giudice, Massimo Borelli, Francesco Bennardo

**Affiliations:** 1 School of Dentistry, Department of Health Sciences, Magna Graecia University of Catanzaro, Viale Europa, 88100 Catanzaro, Italy; selene.barone@unicz.it (S.B.); alessandro.antonelli@unicz.it (A.A.); antonio.madonna@studenti.unicz.it (A.M.); 2 School of PhD Programmes Life Science and Technologies, Magna Graecia University of Catanzaro, Viale Europa, 88100 Catanzaro, Italy; massimo.borelli@unicz.it

**Keywords:** antibiotic prophylaxis, bone-modifying agent, MRONJ, osteonecrosis, review, tooth extraction

## Abstract

**Background/Objectives**: Although several studies have reported antibiotic protocols for the prevention of medication-related osteonecrosis of the jaw (MRONJ) in patients receiving antiresorptive and/or antiangiogenic therapy following tooth extraction, it remains unclear which protocol is the most effective. Accordingly, this scoping review analyzed antibiotic use in dental extractions in these patients, focusing on whether antibiotic duration influences MRONJ occurrence. **Methods**: Two authors independently searched PubMed, Scopus, and Web of Science (2003–2025). Out of the 770 studies screened, 36 were included. Descriptive statistics, a meta-analysis comparing MRONJ incidence in patients treated with high-dose (HD) and low-dose (LD) antiresorptive treatment according to the therapeutic indication and generalized linear mixed models on antibiotic duration were obtained (α = 0.05). **Results**: Amoxicillin, alone or with clavulanic acid, was the most used antibiotic, and in 8 studies it was combined with metronidazole. Seven studies reported parenteral antibiotic administration. Median antibiotic duration was 1.5 days pre-extraction, 5.5 days post-extraction, and 7 days overall. MRONJ risk was significantly higher in HD than LD patients (95% CI: 1.46–5.43; *p* = 0.002), and antibiotic duration was positively associated with reduced MRONJ risk in HD patients (β = –0.15, *p* = 0.026; OR = 0.86, 95% CI: 0.75–0.98). **Conclusions**: The literature shows heterogeneous antibiotic protocols for MRONJ prevention. The increased MRONJ risk and greater protective effect of antibiotics in HD patients suggest that patient risk profile may be more relevant than the antibiotic regimen.

## 1. Introduction

Medication-related osteonecrosis of the jaw (MRONJ) is defined as exposed bone or an intraoral or extraoral fistula in the jawbone persisting for more than 8 weeks in patients who either are currently or were previously treated with bone-modifying agents (BMAs) and/or antiangiogenic drugs, without a history of head and neck radiation therapy or malignancy involving the jaw [1]. BMAs, including bisphosphonates and denosumab, are commonly prescribed to oncological and osteometabolic patients to reduce bone resorption and turnover by inhibiting osteoclastic activity. Currently, BMAs are classified into high-dose and low-dose regimens [2]. High-dose therapy (intravenous bisphosphonates, denosumab 120 mg/4 weeks) is commonly used for the management of bone-metastatic solid tumors and multiple myeloma; low-dose therapy (oral/intramuscular bisphosphonates, zoledronate 5 mg/year, denosumab 60 mg/6 months) is prescribed for osteometabolic conditions, for cancer treatment-induced bone loss, and as adjuvant therapy to prevent cancer recurrence [2,3,4]. In addition to BMAs, antiangiogenic agents, including vascular endothelial growth factor, mammalian target of rapamycin, and tyrosine kinase inhibitors, have been associated with MRONJ in recent years [5,6,7]. These drugs are regarded as high-dose agents, as patients receiving them are at increased risk of developing MRONJ [2]. Accordingly, the incidence of MRONJ is significantly higher in high-dose patients, ranging from 2% to 7%, compared to 0.58% in patients receiving low-dose therapy for osteometabolic conditions [8,9].

MRONJ was first reported in the literature in 2003 and was associated with the use of intravenous bisphosphonates [10]. The development of this condition depends on systemic and oral risk factors, as well as on the dose, duration, and potency of the exposure therapy. Systemic risk factors include uncontrolled diabetes, corticosteroid therapy, and smoking [9]. Regarding oral factors, periodontal disease has been identified as a significant risk, particularly following tooth extraction, which is considered a significant trigger for the occurrence of MRONJ [9,10,11]. However, it remains unclear whether inflammatory factors and periodontal disease are the primary event in the development of the disease or contribute as predisposing factors [11]. In this context, tooth extraction is considered the most appropriate therapeutic approach when dental or periodontal infection is present and conservative or endodontic treatment is not applicable [12].

While low concentrations of oral bisphosphonates may reduce oxidative stress in human periodontal ligament stem cells, which is essential for supporting healing after tooth extraction, the inhibition of bone remodeling and angiogenesis by antiresorptive drugs, combined with risk factors and the terminal mandibular vascularization, limits bone regeneration [13,14,15]. These aspects provide a rationale for the use of systemic antibiotics as a preventive measure in patients at risk of osteonecrosis undergoing dental extractions [14,15]. The use of such agents is also justified by the role that bacterial biofilms and fungal species appear to play in the etiopathogenesis of MRONJ [16].

At present, clinicians face a challenge in choosing the antibiotic protocol for tooth extraction in patients treated with antiresorptive therapy due to the knowledge gap regarding which protocol is most appropriate in reducing the incidence of MRONJ.

This scoping review evaluated the most used antibiotic protocols for patients at risk of MRONJ undergoing tooth extraction, compared MRONJ incidence between patients treated with high- versus low-dose BMAs, and assessed whether the duration of perioperative prophylactic and postoperative therapeutic antibiotic administration affects MRONJ occurrence.

## 2. Results

**Figure 1:**
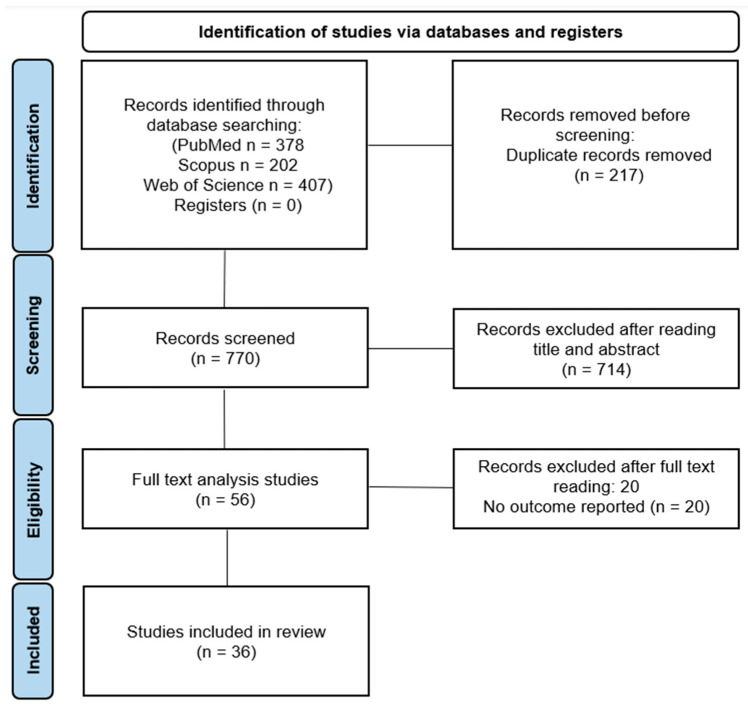
PRISMA flowchart of included and excluded articles.

The details of the literature search are reported in the PRISMA flow diagram (Figure 1).

### 2.1. Study Selection

The search strategy retrieved a total of 987 articles from 3 databases (378 from PubMed, 202 from Scopus, 407 from Web of Science). After removing 217 duplicates, 770 records remained for title and abstract screening. Based on irrelevance to the study objectives, 714 articles were excluded. The full texts of the remaining 56 studies were assessed for eligibility, resulting in the exclusion of 20 articles due to unclear or unreported antibiotic protocol. A total of 36 articles were finally included in the scoping review.

### 2.2. Study Characteristics

Sixteen studies were conducted in Italy [17,18,19,20,21,22,23,24,25,26,27,28,29,30,31,32], 6 studies in Germany [33,34,35,36,37,38], 3 studies in Japan [39,40,41] and Brazil [42,43,44], and 2 studies in the United Kingdom [45,46], Australia [47,48], and Turkey [49,50], and the remaining studies were conducted in the United States [51] and France [52]. These studies were published between 2010 and 2024. Regarding study design, 15 prospective studies [17,18,20,22,23,24,25,26,28,31,33,36,40,47,52], 5 case series [19,21,27,42,44], 10 retrospective studies [29,35,38,39,41,45,48,49,50,51], 1 retrospective case series [30], 1 retrospective cohort study [34], 1 observational study [46], 1 case–control study [43], 1 randomized pilot trial [37], and 1 randomized clinical trial [32] were found. All the data concerning the studies are reported in Table 1.

A total of 5115 patients were analyzed, with study sample sizes ranging from 11 to 759. Data from 30 studies reported a number of extractions ranging from 15 to 1480, with a mean of 295.4 extractions and a median of 171.5. Six studies were excluded from this count, since 2 did not quantify multiple extractions [22,51], 1 referred only to the number of interventions [36], and 3 did not report extraction numbers at all [32,47,52].

A total of 4239 patients and 111 cases of MRONJ or bone exposure were recorded in studies reporting such cases, representing 82.87% of the total sample. The follow-up period ranged from 14 to 3270 days.

Among the included studies, 19 divided patients into high- and low-dose groups, while 7 and 10 studies analyzed outcomes exclusively for the high-dose and low-dose groups, respectively.

The included studies reported the use of different surgical approaches aimed at preventing MRONJ. Notably, a mucoperiosteal flap was raised in 15 studies [17,18,19,21,23,24,27,28,33,35,36,37,38,39,43]. Two of these studies involved elevating a mucoperiosteal flap only in one group to facilitate primary closure, whereas in the other group, no flap was raised and the wound healed by secondary intention [24,36]. Ristow et al. [37] performed a mucoperiosteal flap exclusively in the subperiosteal flap group and compared it to a mucosal flap in the epiperiosteal flap group. Primary or tension-free/mucosal wound closure was another widely adopted approach, reported in 23 studies [17,18,23,24,27,28,31,32,33,34,35,36,37,38,39,40,43,46,48,49,50,51,52].

Flap elevation provided better access to the bone, enabling osteoplasty of the bony edges to be commonly performed following dental extraction in several studies [17,19,20,21,23,24,25,27,28,31,32,33,34,35,37,38,39,43,46,49,50]. In 1 study osteoplasty was only performed in one of two treatment protocols [24]. Two studies reported the use of rotary instruments [17,49], while 5 studies reported the use of piezosurgery [19,20,21,25,28].

Autologous platelet concentrates (APCs) were used in 9 studies [20,23,25,28,29,36,43,46,49]. In one of these, Nd:YAG laser was also employed to enhance healing [49]. In 5 studies, APCs were applied to only one of the two treatment groups [23,29,36,43,46].

Antimicrobial photodynamic therapy (aPDT) with methylene blue and a diode laser was reported in 2 studies [27,42]. One of these also applied low-level laser therapy/photobiomodulation (LLLT/PBM) in cases of postoperative pain or edema [42]. An earlier study employed only LLLT both intraoperatively and postoperatively [26].

Additional strategies for preventing MRONJ included the use of pentoxifylline and tocopherol, which were administered pre- and postoperatively in 1 study [44], as well as O_2_–O_3_ injections, which were applied intra- and postoperatively in another study [32].

**Figure 2:**
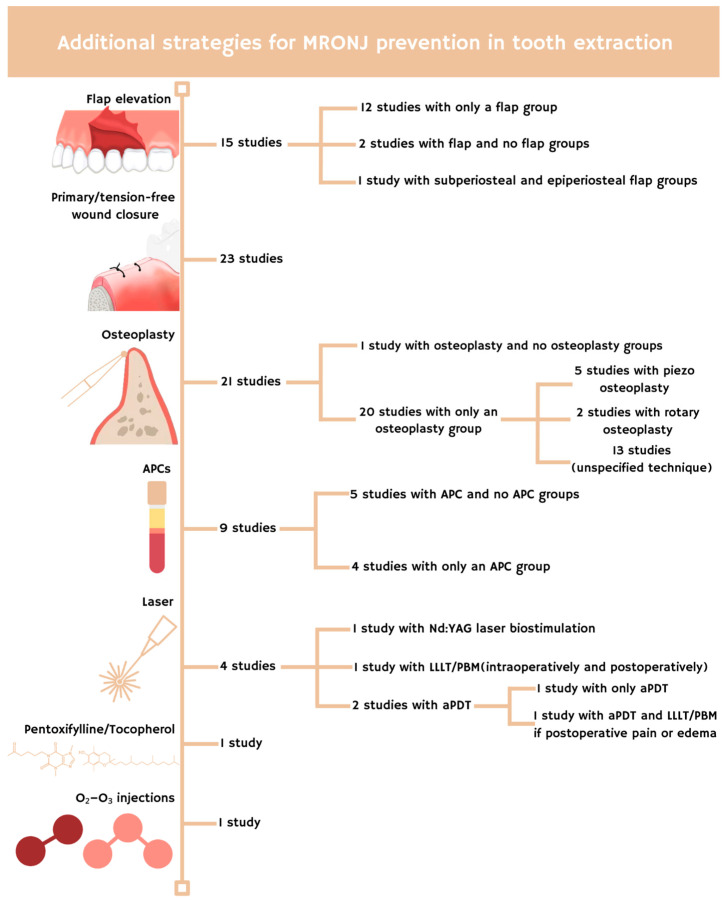
Number of studies presenting additional MRONJ prevention techniques for tooth extractions. APCs: autologous platelet concentrates, LLLT/PBM: low-level laser therapy/photobiomodulation, aPDT: antimicrobial photodynamic therapy.

Figure 2 shows the procedures used to prevent MRONJ during tooth extraction in the included studies.

A total of 12 studies adopted a drug holiday, ranging in duration from three months prior to extraction to three months following the surgical procedure [17,19,21,22,27,31,37,38,41,42,47,50].

Twenty-eight studies reported information about the use of antiseptic treatment aimed at reducing bacterial load [18,19,20,21,22,25,26,27,28,30,31,32,33,35,36,37,38,42,43,44,45,46,47,48,49,50,51,52]. The antiseptic agents used included preoperative and/or postoperative rinses with 0.12–0.2% chlorhexidine, 10% povidone-iodine, hydrogen peroxide (H_2_O_2_), gauze impregnated with 3% H_2_O_2_, and gels containing 1% chlorhexidine or sodium hyaluronate.

### 2.3. Antibiotic Protocol

The most frequently prescribed antibiotic was amoxicillin combined with clavulanic acid (amox-clav), administered at a daily dosage ranging from 2 to 3 g, as reported in 16 protocols from 13 articles [18,19,20,21,23,24,25,26,31,34,38,47,50]. Specifically, 10 protocols from 8 articles used amox-clav at a dosage of 1 g twice a day (BID) for prophylaxis and postoperative therapy [19,21,23,24,26,31,34,50]. One of these studies extended postoperative antibiotic administration until complete mucosal healing was achieved [34].

A prospective study involving 618 patients prescribed amox-clav for prophylaxis only, as a single 2 g dose administered one hour before dental extraction [47]. Three earlier studies reported 3 protocols for prophylaxis and therapy using amox-clav prescribed at 1 g three times a day (TID) [18,20,25]. Finally, a most recent retrospective study that included 759 patients from different treatment groups reported the use of amox-clav at 1 g in 2 protocols for prophylaxis and postoperative therapy, without specifying if it was administered once or multiple times daily [38].

Six protocols, derived from 6 studies, added metronidazole to amox-clav [17,22,28,29,32,49]. Notably, in a prospective study of 12 patients, prophylaxis and postoperative therapy included 1 g of amox-clav BID with the addition of 250 mg of metronidazole TID [22].

In contrast, 2 more recent studies proposed a dosage of 250 mg of metronidazole administered BID for antibiotic prophylaxis and postoperative therapy [28,29]. In the retrospective study involving 37 patients, metronidazole was combined with 1 g of amox-clav BID [29]. In the prospective study, it was combined with 1 g of amox-clav TID [28].

A total daily dose of 500 mg of metronidazole was also proposed by Sahin et al. [49] but administered as a single dose in combination with 1 g of amox-clav once daily, for prophylaxis and postoperative therapy. In a more recent study, Di Fede et al. [32] used 500 mg of metronidazole and 1 g of amox-clav TID, for antibiotic prophylaxis and therapy. Similarly, Saia et al. [17] recommended the same dosages for postoperative therapy only, administering both antibiotics TID for the first three days, followed by BID administration for the subsequent four days.

A total of 10 protocols, reported in 10 studies, used amoxicillin as the sole antibiotic agent [27,30,40,41,42,44,45,48,51,52]. Four of these studies prescribed a dosage of 500 mg TID [42,44,45,51]. Among them, 2 studies administered antibiotics for both prophylaxis and postoperative therapy [42,44]. One study prescribed antibiotics exclusively for postoperative therapy and extended treatment by a further 14 days if an infection was detected during the 14-day follow-up period [51]. The retrospective study involving 652 patients administered antibiotics only to high-risk patients after surgery [45].

Another study administered amoxicillin TID exclusively for postoperative therapy without specifying the dosage [41]. Poli et al. [27] prescribed 1 g of amoxicillin TID for prophylaxis and postoperative therapy, while earlier research administered at least 250 mg of amoxicillin one hour before surgery, followed by a postoperative regimen of 1 g once daily [40].

Two more recent studies used 1 g of amoxicillin BID [30,52]. One of these studies provided prophylaxis only, in the presence of a local infection [52].

Finally, Chang et al. [48] administered 2 g of amoxicillin to high-risk patients one hour before surgery. This study also prescribed postoperative antibiotic therapy for the same group of patients without providing specific details about the dosage, frequency, and duration of administration.

Two studies combined metronidazole with amoxicillin for antibiotic prophylaxis and postoperative therapy [30,43]. Pippi et al. [30] added 500 mg of metronidazole to 1 g of amoxicillin, both administered BID. The more recent case–control study used 400 mg of metronidazole combined with 500 mg of amoxicillin, both prescribed TID [43].

**Figure 3:**
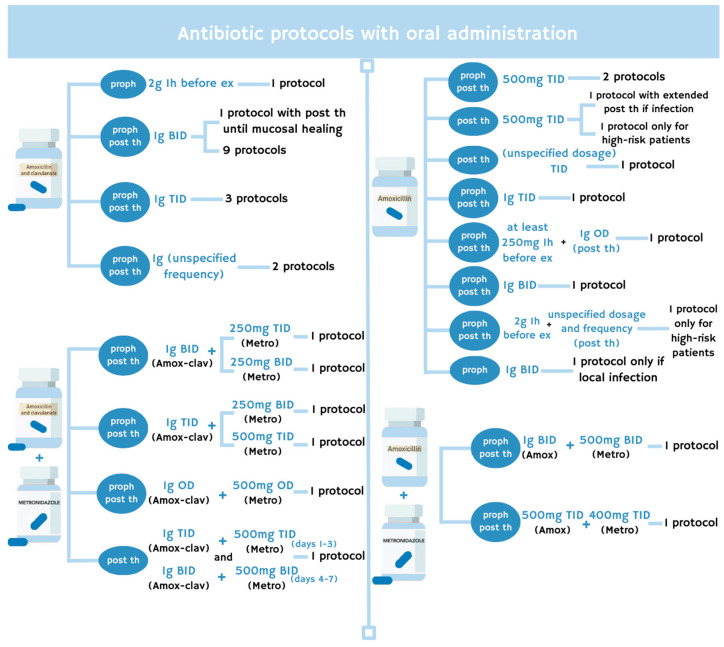
Antibiotic protocols with oral administration reported in the included studies. Proph: antibiotic prophylaxis, post th: postoperative therapy, h: hour, ex: dental extraction, BID: twice a day, TID: three times a day, OD: once a day, amox: amoxicillin, amox-clav: amoxicillin and clavulanic acid, metro: metronidazole.

Figure 3 shows the antibiotic protocols with oral administration described in the included studies.

Seven studies administered broad-spectrum antibiotics via a parenteral route [31,33,35,36,37,38,39]. One protocol from a prospective study for high-risk group included a single 1 g intramuscular dose of ceftriaxone for prophylaxis, followed by a postoperative regimen combining 1 g of ceftriaxone with 1 g of amox-clav BID [31].

Three studies reported the use of intravenous ampicillin and sulbactam for antibiotic prophylaxis and therapy [33,37,38]. Bodem et al. [33] administered 1.5 g of ampicillin and sulbactam TID, while one protocol from a retrospective study for the intravenous group prescribed the same dosage of ampicillin and sulbactam without specifying the dosing frequency [38]. Finally, the randomized pilot trial involving 160 patients used 375 mg of ampicillin and sulbactam BID [37].

Three studies used intravenous administration of 10 million IU of penicillin for both prophylaxis and postoperative therapy [35,36,39]. In cases of purulent infection, Spanou et al. [35] added 500 mg of metronidazole BID to the penicillin regimen.

In the event of an allergy to the antibiotics prescribed in prophylactic and therapeutic protocols, 22 out of 36 studies (61.11%) reported the use of alternative antibiotic regimens [17,20,23,24,25,29,32,33,34,35,36,37,38,39,40,42,44,45,47,48,50,52]. Specifically, 1 study proposed 500 mg of lincomycin BID [17], 4 studies proposed 600 mg of erythromycin TID [20,23,24,25] and 1 study proposed 600 mg of erythromycin combined with 500 mg of metronidazole, both administered TID [32]. Twelve studies used clindamycin, with doses ranging from 150 mg once daily to 600 mg, administered either as a single dose two hours before extraction or two to three times daily [33,34,35,36,37,38,39,42,47,48,50,52]. One study combined 600 mg of clindamycin with 400 mg of metronidazole, both prescribed TID [44]. Finally, metronidazole 400 mg TID was used in 1 study [45], while azithromycin 500 mg and clarithromycin 200–400 mg once daily were each proposed in 1 study [29,40].

The duration of the antibiotic protocol ranged from 1 to 7 days in 22 protocols derived from 21 studies, with both antibiotic prophylaxis and postoperative therapy varying from 0 to 7 days [17,19,20,21,23,24,25,31,32,33,35,36,38,39,40,41,42,45,47,50,52]. In contrast, antibiotics were administered for more than 7 days in 17 protocols derived from 14 studies, with prophylaxis ranging from 0 to 14 days and postoperative therapy ranging from 5 to 20 days [18,22,26,27,28,29,30,31,37,38,43,44,49,51]. One study has not included either antibiotic prophylaxis or postoperative therapy [46]. Two additional studies were excluded from the total duration count because the duration of postoperative therapy could not be quantified: the retrospective cohort study of 72 patients prescribed antibiotics until mucosal healing [34], while the retrospective study by Chang et al. [48] has not provided details regarding postoperative therapy.

### 2.4. Data Analysis

Thirty-six studies reported data on prophylactic antibiotic protocols, with 3 studies including more than 1 protocol, resulting in a total of 42 distinct prophylactic regimens. Each protocol was considered individually in the analysis. The mean duration of antibiotic prophylaxis was 2.36 ± 2.71 days, with a median of 1.5 days.

Thirty-four studies provided detailed data on prophylactic and therapeutic antibiotic protocols, resulting in 40 distinct antibiotic protocols. The mean duration of postoperative antibiotic therapy was 6.52 ± 4.77 days, with a median of 5.5 days. When both prophylactic and therapeutic regimens were considered, the mean duration of antibiotic administration was 8.98 ± 5.85 days, with a median of 7 days.

**Figure 4:**
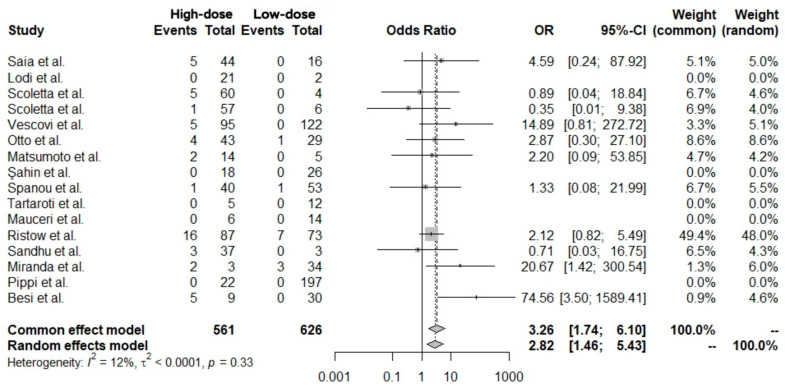
Random effects and meta-analysis comparing MRONJ cases (events) between high-dose (experimental) and low-dose (control) treatment groups [17,18,20,25,26,28,29,30,34,35,37,39,42,46,49,51]. OR: Odds Ratio, 95%-CI: 95% Confidence Interval.

A conventional meta-analysis of 16 studies directly comparing high- versus low-dose treatment groups, including 1187 patients (561 receiving high-dose therapy and 626 receiving low-dose therapy), was performed. Overall, 61 cases of MRONJ were observed. The pooled analysis showed that patients receiving high-dose treatment had a significantly higher risk of developing MRONJ compared with those receiving low-dose treatment (Figure 4).

The common-effect model yielded an Odds Ratio (OR) of 3.26 (95% CI: 1.74–6.10; *p* = 0.0002; Table 2), while the random-effects model estimated an OR of 2.82 (95% CI: 1.46–5.43; *p* = 0.002; Table 2). Between-study heterogeneity was low (I^2^ = 12.0%, 95% CI 0.0–52.3%; τ^2^ < 0.0001) and not statistically significant (Q = 11.36, df = 10, *p* = 0.33). Given the presence of potential clinical and methodological variability across studies, the random-effects model was considered the primary estimate.

In parallel, to investigate whether the duration of antibiotics influenced the incidence of MRONJ, we fitted generalized linear mixed models (GLMMs) with a binomial distribution and logit link, including random intercepts for study, based on a total of 32 studies that clearly reported both the number of MRONJ cases and the duration of prophylactic and postoperative therapeutic regimens. In these 32 studies, sample sizes ranged from 3 to 700 patients per study arm (median 41.5). The number of MRONJ cases varied from 0 to 23 per study (mean 2.5), with a higher frequency in high-dose groups. In the high-dose arms, sample sizes ranged from 0 to 180 patients (mean 27.6), with MRONJ cases ranging from 0 to 16 (mean 2.0). The low-dose arms included up to 700 patients (mean 70.0), with fewer MRONJ cases on average (mean 0.5, maximum 7). The reported duration of antibiotic administration ranged from 0 to 23 days, with a mean of 9 days and a median of 7 days. Two nested models were compared: Model 1 included fixed effects for antibiotic duration and dose group (high vs. low), while Model 2 additionally included the interaction between duration and dose. Model comparison using AIC (149.1 vs. 145.8) and likelihood ratio testing (χ^2^ = 5.28, df = 1, *p* = 0.022) favored Model 2, which was therefore retained as the final model. The results of the final model are shown in Table 3.

**Figure 5:**
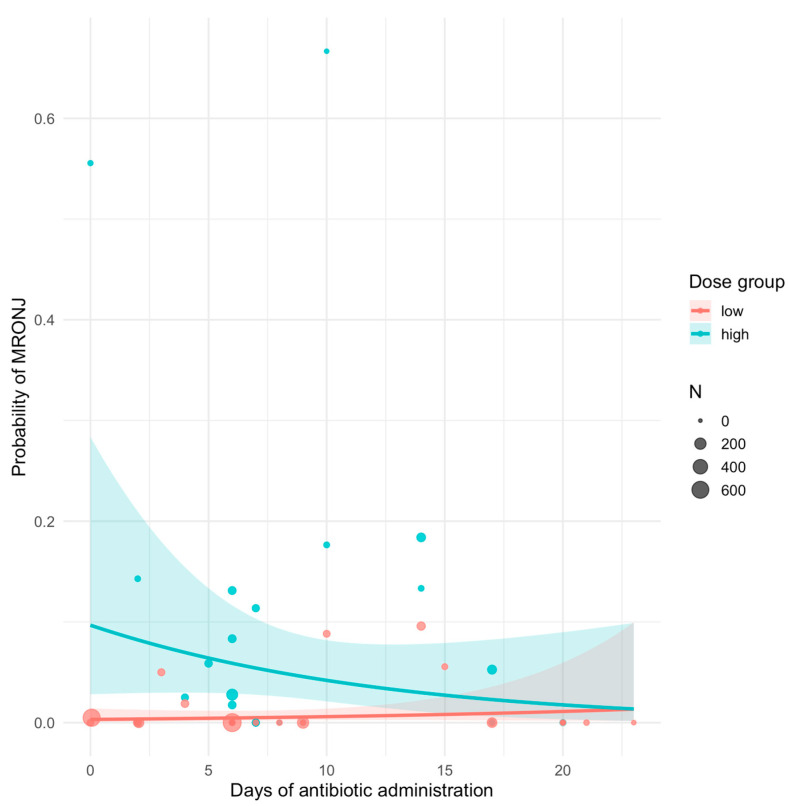
R plot showing the relationship between antibiotic duration and MRONJ risk in high- and low-dose groups. Each point represents study-level data weighted by sample size; shaded areas indicate 95% confidence intervals.

High-dose therapy was strongly associated with increased risk of MRONJ (β = +3.51, *p* < 0.001; OR ≈ 33.5). In the low-dose group, longer antibiotic duration showed a null trend toward lower MRONJ risk (β = +0.06 per day, *p* = 0.35; OR ≈ 1.06; Figure 5). In contrast, in the high-dose group the interaction term indicated a significant protective effect of longer antibiotic duration, with each additional day of therapy associated with a 14% reduction in the odds of MRONJ (β = –0.15, *p* = 0.026; OR = 0.86, 95% CI: 0.75–0.98; Figure 5). The between-study variance was estimated at 1.85 (SD = 1.36), confirming heterogeneity across studies, although the main fixed effects remained statistically robust.

## 3. Materials and Methods

### 3.1. Study Design

This scoping review was conducted in accordance with the PRISMA Extension for Scoping Reviews (PRISMA-ScR): Checklist and Explanation guidelines [53]. This study followed a different methodology than a systematic review, focusing on a more inclusive approach to examine how antibiotic protocols are used to prevent MRONJ after tooth extraction, rather than restricting study types or performing a risk of bias and quality assessment of the included studies.

### 3.2. PICO Question

“Is there an antibiotic regimen that is effective in reducing the incidence of MRONJ in patients undergoing tooth extraction while receiving bone-modifying and/or antiangiogenic agents, compared with protocols in which other antibiotics or no antibiotics are used?”

### 3.3. Search Strategy

Medline (using PubMed), Scopus, and Web of Science databases were electronically searched from 2003 to 2025, restricted to studies with abstracts in English. The search strings adopted for each database are detailed in Table 4.

### 3.4. Inclusion and Exclusion Criteria

The following inclusion criteria were applied: (1) original articles published in English language, (2) studies involving human participants, (3) studies with a sample size of at least 10 patients, (4) studies involving patients who underwent tooth extraction with antibiotic administration, and (5) studies including patients treated with BMAs and/or antiangiogenic drugs.

The following exclusion criteria were applied: (1) studies conducted in vitro, (2) studies involving animal models, (3) studies including patients with history/ongoing MRONJ, (4) studies involving patients with history of head and neck radiation, and (5) literature reviews, case reports, letters, editorials, doctoral theses, or abstracts.

The reference lists of the included studies were analyzed to complement the electronic search strategy with manual search.

### 3.5. Selection of the Studies

The manuscripts selected included case series, randomized clinical trials, randomized pilot trials, retrospective studies, prospective studies, and observational studies. Two investigators (FB, AM) independently performed the database searches. Disagreements were resolved in a consensus meeting with a third reviewer (AG).

### 3.6. Data Extraction

Duplicates obtained from the search strategy were identified and excluded using Rayyan QCRI (Rayyan Systems Inc., Cambridge, MA, USA) [54]. Data extraction was performed separately by two authors (FB, AM), and discrepancies were subject to consultation with a third reviewer (AG).

The following data were extracted from the studies: authors, publication date, study design, number of patients and gender distribution, age, number and sites of tooth extractions, treatment type (high-dose, low-dose) and underlying pathology (oncologic, osteometabolic), antibiotic prophylaxis and postoperative antibiotic therapy (type, dose, duration, route), antiseptic use, additional prevention strategies, and number of MRONJ/bone exposure cases (including follow-up time and treatment type).

The duration of antibiotic prophylaxis and postoperative antibiotic therapy was recorded as whole numbers of days. When antibiotics were administered one hour prior to tooth extraction, the duration was counted in decimal form (e.g., 0.05 days).

### 3.7. Statistical Analysis

Summary statistics and dataset structure were first examined to ensure data integrity and suitability for subsequent modeling. Descriptive statistics were reported as medians, means, and standard deviations for continuous quantitative variables, and as absolute and relative frequencies for categorical data.

The primary outcome was the incidence of MRONJ, expressed as the number of cases over the total number of patients within each study arm. When available, two treatment arms, a high-dose and a low-dose group, were considered for each study.

A meta-analysis was conducted to compare the incidence of MRONJ in patients receiving high-dose versus low-dose treatment. Odds ratios (ORs) with 95% confidence intervals (CIs) were calculated. Both a common-effect model (Mantel–Haenszel method) and a random-effects model (inverse-variance method with restricted maximum likelihood estimation of between-study variance) were fitted. Between-study heterogeneity was assessed using Cochran’s Q statistic, the I^2^ statistic, and the estimate of τ^2^ with 95% confidence intervals. The random-effects model was considered the primary analytical approach given the potential for clinical and methodological variability across studies.

To assess whether the duration of antibiotic administration influenced MRONJ incidence, we fitted generalized linear mixed models (GLMMs) with a binomial distribution and logit link, using the lme 4 library [55]. The models were specified at the arm level, with the number of MRONJ events as the numerator and the total number of patients as the denominator, and included random intercepts for study to account for between-study heterogeneity.

Two models were compared. The first was a baseline model, which included fixed effects for antibiotic duration (in days) and dose group (high vs. low). The second was an extended model, which additionally included the interaction between antibiotic duration and dose group. Model comparison was performed using the Akaike Information Criterion (AIC) and likelihood ratio tests.

The final model was selected based on these criteria, and parameter estimates were reported as regression coefficients(β) with standard errors, z-values, and *p*-values. ORs with 95% CIs were derived for interpretability. All statistical analyses were conducted using R software (version 4.4.1; R Foundation for Statistical Computing, Vienna, Austria), with a two-sided significance level of α = 0.05 [56].

## 4. Discussion

This scoping review aimed to examine the literature on antibiotic protocols before and after dental extractions for patients at risk of MRONJ, using a meta-analysis to evaluate the difference in MRONJ incidence between patients treated with high- versus low-dose BMAs and a generalized linear mixed model to assess the potential impact of antibiotic duration on the occurrence of MRONJ.

To date, the pathogenesis of MRONJ still needs to be clarified, with the most widely accepted hypothesis involving reduced bone turnover due to the inhibition of osteoclast activity by antiresorptive agents, leading to the accumulation of microfractures. However, other factors, such as infection and inflammation, should be considered [57]. Notably, dental extraction facilitates bacterial invasion in the oral cavity, whose microbiome is particularly susceptible to external pathogens than other regions of the digestive tract, thereby promoting the development of infectious processes [58,59]. Evidence also suggests that the presence of periapical or periodontal infections may impair osteoclast function and increase the risk of MRONJ, even when tooth extraction is not performed [58].

Moreover, MRONJ may be related to an imbalanced oral microbiota, as bisphosphonates can increase bacterial adhesion to bone and impair the local microbial environment [58,59]. For these reasons, to reduce the risk of developing MRONJ, tooth extraction in patients undergoing antiresorptive therapy requires the careful implementation of preventive strategies, such as antibiotic administration.

In an attempt to identify the etiological role of bacteria in MRONJ, research has revealed that MRONJ lesions have a different microbial profile than unaffected areas [58,60,61]. A recent study has identified an increased abundance of the phylum Bacteroidetes in patients affected by MRONJ, a bacterial group known to impair wound healing [60]. At the genus level, multiple studies have reported a higher prevalence of Pyramidobacter, Prevotella, Porphyromonas, Actinomyces, and Fusobacterium in samples collected from necrotic bone [58,60,61]. Actinomyces is frequently detected in the submucosal layer, where it helps establish an acidic and anaerobic environment that can protect against antibiotics [60,61]. Dental procedures may further promote such acidic conditions and, together with the metabolic activity of *Streptococcus* spp., may predispose to the development of MRONJ [58,60].

In the evaluation of antibiotic resistance in bacteria-associated MRONJ, the first-line treatment for Actinomyces and other bacterial groups implicated in MRONJ lesions appears to be β-lactam agents plus β-lactamase inhibitors [61]. A literature review confirms that clinicians tend to prefer penicillin-based antibiotics with β-lactamase inhibitors and metronidazole for the treatment of MRONJ, rather than penicillin alone or alternative regimens [62]. The combination of full-dose penicillin and metronidazole is supported by the 2024 position paper of the Italian Societies of Oral Pathology and Medicine and Maxillofacial Surgery (SIPMO-SICMF position paper), which recommends a duration of 7 to 14 days for MRONJ patients [2]. While this represents an antibiotic therapy used for the treatment of MRONJ, there is no consensus among authors regarding the optimal antibiotic regimen and timing of prophylaxis and postoperative therapy for preventing MRONJ in patients undergoing dental extraction.

Currently, in oral clinical practice, antibiotics are empirically prescribed, usually as broad-spectrum treatments for short periods not exceeding 7–10 days [63]. However, their use should be limited to invasive procedures in patients at risk of bacterial endocarditis, as well as those predisposed to infection, such as oncologic, immunocompromised, and uncontrolled diabetic patients [64,65]. In this study, the median total duration of antibiotic administration was 7 days, including 1.5 days preoperatively and 5.5 days postoperatively. This finding is consistent with the minimum recommended antibiotic duration for MRONJ patients.

Regarding antibiotic class, the data collected in this review identified the use of amox-clav in 38.1% of 42 protocols, while 23.8% used amoxicillin alone. Eight protocols (19.0%) associated amox-clav or amoxicillin with metronidazole. This pattern confirms the preference for β-lactam antibiotics, often combined with a β-lactamase inhibitor, as the antibiotic protocol of choice in the prevention of MRONJ. Conversely, the combination of amox-clav or amoxicillin with metronidazole could be justified, as their synergistic effect allows the antimicrobial activity to occur at concentrations below the Minimum Inhibitory Concentration (MIC) of the target anaerobic bacteria [66].

In this review, three out of 8 protocols administered amoxicillin or amox-clav in combination with 400/500 mg of metronidazole TID [17,32,43]. This higher dosage may be justified, as the combination of amoxicillin with 400/500 mg of metronidazole TID has shown significant efficacy against periodontopathogens similar to those implicated in the development of MRONJ, when compared to lower dosages [67,68].

Focusing on amox-clav, ten out of 16 protocols prescribed a dosage of 1000 mg BID [19,21,23,24,26,31,34,50]. This regimen ensures plasma concentrations above the MIC of bacteria commonly involved in orofacial odontogenic infections [69]. The lower cumulative dose of clavulanic acid, compared with an 8 h dosing interval, also improves patient compliance and reduces the risk of gastrointestinal adverse effects [69].

However, defining a proper dosing regimen for broad-spectrum antibiotics is challenging, since their pharmacokinetics can be altered by several factors, potentially reducing therapeutic efficacy or increasing the risk of toxicity [70,71]. This is particularly relevant for elderly patients undergoing BMAs, as age-related cardiovascular changes can affect the drug volume of distribution [70]. Specifically, increased capillary permeability due to systemic inflammation and continuous fluid infusion, may increase the volume of distribution of hydrophilic antibiotics such as beta-lactams, making a loading dose necessary. On the other hand, an increase in body fat and the subsequent decrease in total body water may contract the volume of distribution of hydrophilic antibiotics and increase that of lipophilic drugs [70].

A decline in renal function can also affect the pharmacokinetics of antibiotics used to prevent MRONJ after tooth extraction, as most of these are eliminated through the kidneys. While metronidazole usually does not require dose adjustment, penicillins may need dose modification in cases of severe renal dysfunction, despite their favorable safety profile [71].

The current literature reports several additional preventive approaches for MRONJ in the context of dental extraction. Mucoperiosteal flap elevation is a widely employed technique, as it provides adequate visibility for surgery and minimizes soft tissue trauma during tooth extraction [72]. In this regard, a wide flap base ensures an adequate blood supply and may reduce the risk of developing MRONJ [72,73].

In cases of mucosal disruption, tension-free primary wound closure may help prevent impaired healing and postoperative complications [72,73]. However, Malden et al. [74] reported that while primary wound closure is not crucial, smoothening sharp bony edges, without separating the periosteum from the underlying bone, is essential. Alveoloplasty was performed in 21 out of 36 studies (58%), supporting its usefulness in reducing the risk of irritation and necrotic bone formation, particularly in patients at high risk of MRONJ [72].

An additional preventive approach involves the use of APCs, which are known to promote soft tissue healing by supporting angiogenesis through platelet-derived growth factors. While in vitro and animal model studies emphasize the potential role of APCs in preventing MRONJ, a review showed no significant impact on the occurrence of MRONJ after tooth extraction [12].

LLLT/PBM with Nd:YAG laser was used in two studies for its antimicrobial properties and ability to reduce oxidative stress, which may accelerate surgical wound healing [26,42,75]. One study successfully reported the combination of APCs and Nd:YAG laser [49]. Furthermore, Tartaroti et al. combined LLLT/PBM with aPDT, which may be useful for preventing MRONJ due to its antimicrobial activity resulting from the generation of reactive oxygen species [42].

Interestingly, another non-invasive technique for the prevention of MRONJ involves the use of two pharmacological agents, pentoxifylline and tocopherol. The effectiveness of this approach may be attributed to the interaction between pentoxifylline’s ability to enhance tissue perfusion and tocopherol’s antioxidant properties [76].

The risk of MRONJ could also be reduced by using O_2_-O_3_ injections, as they have been shown to promote clinical healing of the post-extraction site when used as an adjunctive therapy [32].

Ultimately, among the various approaches described in the literature for the prevention of MRONJ, drug holiday has been reported [74]. This strategy remains controversial and should not replace other preventive measures, such as atraumatic extractions and antibiotic prophylaxis. A drug holiday may be useful in the case of denosumab, due to its short half-life, whereas a short-term interruption of bisphosphonates may not appear to reduce the risk of MRONJ significantly. Indeed, bisphosphonates have a residual effect that persists for many years after treatment discontinuation because the drug remains bound to the bone [74]. Despite the lack of evidence supporting a bisphosphonate prophylactic drug holiday before tooth extraction, the SIPMO-SICMF position paper recommends discontinuing bisphosphonates one week before surgery until complete mucosal healing is achieved [2]. If authorized by the prescribing clinician, discontinuation is recommended for patients on high-dose therapy. For patients under treatment with low-dose therapy, a drug holiday is recommended if under treatment for more than three years or in case of systemic risk factors. This strategy could thus reduce antiangiogenic activity, oral mucosal toxicity, and the inhibition of epithelial cell proliferation and migration after tooth extraction [2,74].

The results of this scoping review may help clinicians define antibiotic protocols for patients at risk for MRONJ undergoing tooth extraction. Specifically, the use of amoxicillin/amoxicillin–clavulanic acid alone or in combination with metronidazole may be reasonable, as it represents the most common antibiotic regimen. Furthermore, the median total duration of 7 days found in this review may be considered appropriate, as it is consistent with the duration of antibiotic therapy typically prescribed for oral surgery procedures, including surgical treatment of MRONJ.

This review has several strengths. To our knowledge, no previous analyses have examined the relationship between the duration of antibiotic administration and the incidence of MRONJ in patients receiving BMAs and/or antiangiogenetic agents undergoing tooth extraction. The review included 36 studies and over 5000 patients, considering both high- and low-dose therapies and various antibiotic regimens, thus providing a broad overview of current clinical practice. Furthermore, data extraction and statistical analyses allowed a quantitative evaluation of the potential association between antibiotic duration and MRONJ occurrence.

However, the present study’s limitation is that its reliance on aggregated (study-level) data precludes patient-level analyses that could adjust for individual confounders and allow for more robust modeling. Consequently, the inferential results should be interpreted with caution.

## 5. Conclusions

The literature highlights considerable heterogeneity in antibiotic protocols for MRONJ prevention. Likely due to the higher overall risk in high-dose patients, our findings suggest that maintaining an appropriate duration of the antibiotic regimen in this group may help prevent MRONJ following tooth extraction. Few MRONJ cases were observed regardless of the antibiotic regimen, suggesting that the patient’s overall risk profile may play a more significant role than the specific antibiotic protocol. Future multicenter studies with larger sample sizes and standardized protocols are needed to clarify the role of antibiotics in patients at risk of MRONJ undergoing tooth extraction.

## Figures and Tables

**Table 1:** Main characteristics of the included studies.

Reference	Study Sample (M-F)	Mean/ Median Age	Extraction (n; Site)	Low and High-Dose Treatment (n Patients)	Causes for Treatment (n Patients)	Antibiotic Prophylaxis in Tooth Extraction	Antibiotic Therapy in Tooth Extraction	Antiseptic Treatment	Additional Strategies for MRONJ Prevention in Tooth Extraction	n MRONJ/Bone Exposure Cases at Follow-Up end (Follow-Up Time; Treatment Type)	
Saia et al., 2010 [17] Prospective study	60(18-42)	65 yrs	185 ex 103 ex mdb 82 ex max	High-dose (44; 8 patients received both Z and P) Low-dose (16)	Oncologic (44) Osteometabolic (16)	No antibiotic prophylaxis	Days 1–3: Amox-clav 1 g TID × 3 d + Metro 500 mg TID × 3 d Days 4–7: Amox-clav 1 g BID × 4 d + Metro 500 mg BID × 4 d *If allergy:* Linco 500 mg BID × 7 d	N/A	Mucoperiosteal flap, osteoplasty with rotary instruments, tension-free wound closure Drug holiday: 1 month after ex	5 (6 months; HD)	
Lodi et al., 2010 [18] Prospective study	23 (18-42)	68.2 yrs	38 ex Interventions: 23 mdb 5 max 2 both	High-dose (21) Low-dose (2)	Oncologic (21) Osteometabolic (2)	Amox-clav 1 g TID × 3 d	Amox-clav 1 g TID × 17 d	*Since ex decision:* 0.2% CHX rinse OD *Postop:* 1% CHX gel TID × 14 d	Mucoperiosteal flap, primary wound closure	0 (mean: 229.5 d, range: 14–965 d)	
Ferlito et al., 2010 [19] Case series	34 (N/A)	53.8 ± 4.2 yrs	71 ex 31 ex mdb 40 ex max	High-dose (34)	Oncologic (34)	Amox-clav 1 g BID × 2 d	Amox-clav 1 g BID × 5 d	*Postop:* 0.2% CHX or 10% povidone-iodine rinse	Mucoperiosteal flap, osteoplasty with piezosurgery/rongeur Drug holiday: 13 patients	0 (12 months)	
Scoletta et al., 2011 [20] Prospective study	64 (20-45)	64.81 ± 10.98 yrs	220 ex 113 ex mdb 107 ex max	High-dose (60; 7 patients received both Z and P) Low-dose (4)	Oncologic (60) Osteometabolic (4)	Amox-clav 1 g TID × 1 d *If allergy:* Erythro 600 mg TID × 1 d	Amox-clav 1 g TID × 5 d *If allergy:* Erythro 600 mg TID × 5 d	*Preop:* 0.12% CHX rinse BID × 7 d *Postop:* 3% H_2_O_2_ gauze TID × 14 d	Ostectomy and/or osteoplasty with piezosurgery, PRF	5 (mean: 13.06 ± 1.35 months, range: 4–24 months; HD)	
Ferlito et al., 2011 [21] Case series	43 (N/A)	56.4 ± 5.8 yrs	102 ex 43 ex mdb 59 ex max	High-dose (43)	Oncologic (43)	Amox-clav 1 g BID × 2 d	Amox-clav 1 g BID × 5 d	*Postop:* 0.2% CHX or 10% povidone-iodine rinse	Mucoperiosteal flap, osteoplasty with piezosurgery/rongeur Drug holiday: 17 patients	0 (12 months)	
Carini et al., 2012 [22] Prospective study	12 (N/A)	N/A	18 ex 2 full-mouth ex	Low-dose (12)	N/A	Amox-clav 1 g BID × 14 d + Metro 250 mg TID × 14 d	Amox-clav 1 g BID × 7 d + Metro 250 mg TID × 7 d	*Preop:* 0.2% CHX single rinse *Postop:* 0.2% CHX rinse × 7 d	*If low-dose < 3 yrs + risk factors or low-dose > 3 yrs:* MD consult for 3 months drug holiday pre/postop	0 (12 months)	
Mozzati et al., 2012 [23] Prospective study	*Study group:* 95 (36-55)	Range: 44–83 yrs	275 ex 142 ex mdb 133 ex max	High-dose (180)	Oncologic (180)	Amox-clav 1 g BID × 1 d *If allergy:* Erythro 600 mg TID × 1 d	Amox-clav 1 g BID × 5 d *If allergy:* Erythro 600 mg tid × 5 d	N/A	Mucoperiosteal flap, osteoplasty, PRF, primary wound closure	0 (range: 24–60 months)	
	*Control group:* 85 (39-46)		267 ex 145 ex mdb 122 ex max						Mucoperiosteal flap, osteoplasty, primary wound closure	5 (range: 24–60 months; HD)	
Mozzati et al., 2013 [24] Prospective study	*Protocol A:* 334 (15-339)	Range: 52–79 yrs	620 ex 368 ex mdb 252 ex max	Low-dose (700)	Osteometabolic (700)	Amox-clav 1 g BID × 1 d *If allergy:* Erythro 600 mg TID × 1 d	Amox-clav 1 g BID × 5 d *If allergy:* Erythro 600 mg TID × 5 d	N/A	Mucoperiosteal flap, osteoplasty, primary wound closure	0 (range: 12–72 months)	
	*Protocol B:* 366 (8-358)		860 ex 496 ex mdb 364 ex max						Socket filled with absorbable gelatin sponge, secondary intention		
Scoletta et al., 2013 [25] Prospective study	63 (18-45)	65.82 yrs	202 ex 111 ex mdb 91 ex max	High-dose (57) Low-dose (6)	Oncologic (57) Osteometabolic (6)	Amox-clav 1 g TID × 1 d *If allergy:* Erythro 600 mg TID × 1 d	Amox-clav 1 g TID × 5 d *If allergy:* Erythro 600 mg TID × 5 d	*Postop:* 3% H_2_O_2_ gauze tid × 14 d	Osteoplasty with piezosurgery, PRF	1 (range: 4–12 months; HD)	
Vescovi et al., 2013 [26] Prospective study	217 (38-179)	68.72 ± 11.26 yrs	589 ex 285 ex mdb 304 ex max	High-dose (95) Low-dose (122)	Oncologic (95) Other (122)	Amox-clav 1 g BID × 3 d	Amox-clav 1 g BID × 14 d	*Postop:* CHX + H_2_O_2_ rinse until healing	Socket irrigation with povidone-iodine solution and LLLT application PBM weekly × 6 wks + additional treatments until healing	5 (mean: 15 months, range: 4–31 months; HD)	
Hutcheson et al., 2014 [47] Prospective study	618 (N/A)	N/A	N/A	Low-dose (618)	Osteometabolic (618)	Amox-clav 2 g 1 h preop *If allergy:* Clinda 600 mg 1 h preop	No postoperative therapy	*Postop:* 10 mL CHX rinse TID × 7 d	*If CTX < 150 pg/mL:* drug holiday	3 (minimum FU 2 months; LD)	
Bodem et al., 2015 [33] Prospective study	61 (19-42)	66.65 ± 12.69 yrs	184 ex 47 ex mdb 55 ex max	High-dose (61)	Oncologic (61)	Amp-sulb IV 1.5 g TID × 1 d *If allergy:* Clinda 600 mg TID × 1 d	Amp-sulb IV 1.5 g TID × 5 d *If allergy:* Clinda 600 mg TID × 5 d	0.12% CHX rinse TID	Mucosal flap if requested, osteotomy if requested, tension-free closure	8 (3 months; HD)	
Otto et al., 2015 [34] Retrospective cohort study	72 (19-53)	67.5 yrs	216 ex 97 ex mdb 119 ex max	High-dose (43) Low-dose (29)	Oncologic (43) Osteometabolic (29)	Amox-clav 1 g BID × 1 d *If allergy:* Clinda 600 mg TID × 1 d	Amox-clav 1 g BID until mucosal healing *If allergy:* Clinda 600 mg TID until mucosal healing	N/A	Atraumatic ex, osteoplasty, mucosal wound closure	5 (mean: 14.9 months; 4 HD, 1 LD)	
Matsumoto et al., 2017 [39] Retrospective study	19 (6-13)	69.3 yrs	40 ex 19 ex mdb 21 ex max	High-dose (14) Low-dose (5)	Oncologic (14) Osteometabolic (5)	Penic IV 10M U.I. x 1 d *If allergy:* Clinda 600 mg TID × 1 d	Penic IV 10M U.I: x 1 d If allergy: Clinda 600 mg TID × 1 d	N/A	Mucoperiosteal flap, osteoplasty, primary wound closure	2 (minimum FU 3 months; HD)	
Shudo et al., 2018 [40] Prospective study	132 (20-112)	71.9 ± 11.4 yrs	274 ex 109 ex mdb 165 ex max	Low-dose (132)	Osteometabolic (77) Osteoporosis prevention (55)	Amox ≥ 250 mg 1 h preop or Clarithro ≥ 200 mg 1 h preop	Amox 1 g OD up to 2 d or Clarithro 400 mg OD up to 2 d	N/A	Atraumatic ex, tension-free wound closure	0(minimum FU 3 months)	
Poli et al., 2019 [27] Case series	11 (3-8)	72.5 ± 4.2 yrs	62 ex 35 ex mdb 27 ex max	Low-dose (11)	Osteometabolic (11)	Amox 1 g TID × 3 d	Amox 1 g TID × 17 d	*Preop:* 0.2% CHX rinse bid × 14 d *Postop:* 0.2% CHX rinse BID × 14 d	Mucoperiosteal flap, osteoplasty, aPDT with methylene blue + diode laser, primary wound closure Drug holiday: 2 months before ex until healing if BPs < 3yrs (4 patients)	0 (range: 6–12 months)	
Şahin et al., 2020 [49] Retrospective study	44 (12-32)	66.3 yrs	63 ex 40 ex mdb 23 ex max	High-dose (18) Low-dose (26)	Oncologic (21) Osteometabolic (23)	Amox-clav 1 g OD × 3 d + Metro 500 mg OD × 3 d	Amox-clav 1 g OD × 14 d + Metro 500 mg OD × 14 d	*Preop:* 0.12% CHX rinse × 3 d *Postop:* 0.12% CHX rinse × 14 d	Sulcular incision if requested, osteoplasty with burs, PRF + Nd: YAG laser biostimulation on 2, 5, 7, 10, 14, 21, and 28 d, primary wound closure	0 (mean: 14.2 months, range: 6–28 months)	
Spanou et al., 2020 [35] Retrospective study	84 (17-67)	71.7 yrs	232 ex 125 ex mdb 107 ex max	High-dose (40) Low-dose (53; 18 patients received two different BPs)	Oncologic (39) Osteometabolic (44) Other (1)	Penic IV 10M U.I. × 1 d + *If purulent infection:* Metro 500 mg bid × 1 d *If allergy:* Clinda 600 mg tid × 1 d	Penic IV 10M U.I. × 3 d + *If purulent infection:* Metro 500 mg bid × 3 d *If allergy:* Clinda 600 mg tid × 1 d	*Preop:* CHX single rinse *Postop:* CHX rinse ≥ 2–3 wks until mucosal healing	Mucoperiosteal flap, osteoplasty, primary wound closure	2 (mean: 41.5 ± 29.5 months, range: 2–109 months; 1 HD, 1 LD)	
Tartaroti et al., 2020 [42] Case series	17 (0-17)	68.94 ± 11.72 yrs	37 ex 20 ex mdb 17 ex max	High-dose (5) Low-dose (12)	Oncologic (5) Osteometabolic (12)	Amox 500 mg TID × 1 d *If allergy:* Clinda 300 g TID × 1 d or Amp 400 mg BID × 1 d	Amox 500 mg TID × 6 d *If allergy:* Clinda 300 g TID × 6 d or Amp 400 mg BID × 6 d	*Preop:* 0.12% CHX rinse *Postop:* 0.12% CHX rinse	aPDT with methylene blue + diode laser, repeated weekly until healing If pain/edema postop: PBM therapy Drug holiday: 6 patients	0 (minimum FU 6 months)	
Mauceri et al., 2020 [28] Prospective study	20 (4-16)	72.35 ± 7.19 yrs	63 ex 30 ex mdb 33 ex max	High-dose (6) Low-dose (14)	Oncologic (6) Osteometabolic (14)	Amox-clav 1 g TID × 1 d + Metro 250 mg BID × 1 d	Amox-clav 1 g TID × 7 d + Metro 250 mb BID × 7 d	*Preop:* 0.2% CHX rinse tid × 7 d *Postop:* 0.2% CHX rinse tid × 15 d + Na-hyaluronate gel tid × 10 d	Mucoperiosteal flap, osteoplasty with piezosurgery, PRP, tension-free wound closure	0 (minimum FU 24 months)	
Poxleitner et al., 2020 [36] Prospective study	Primary closure group: 39 (0-39)	77 yrs	Interventions: 19 mdb 13 max 7 both	Low-dose (77)	Osteometabolic (77)	Penic IV 10M U.I. × 1 d *If allergy:* Clinda 600 mg TID × 1 d	Penic IV 10M U.I. × 1 d *If allergy:* Clinda 600 mg TID × 1 d	*Preop:* CHX single rinse *Postop:* CHX rinse until healing	Sulcular incision for atraumatic ex, mucoperiosteal flap, primary tension-free closure	0 (3 months)	
	PRF group: 38 (1-37)	78 yrs	Interventions: 20 mdb 16 max 2 both						Sulcular incision for atraumatic ex, PRF without primary closure		
Lesclous et al., 2020 [52] Prospective study	44 (0-44)	70 yrs	N/A	Low-dose (44)	Osteometabolic (44)	*If local infection* (13 patients): Amox 1 g BID × 6–7 d *If local infection + allergy:* Clinda 600 mg BID × 6–7 d	No postoperative therapy	*Postop:* 0.2% CHX rinse TID × 7 d	Atraumatic ex, tension-free wound closure and full-closure not mandatory	0 (3 months)	
Sandhu et al., 2020 [51] Retrospective study	40 (20-20)	65 yrs	26 single ex 14 multiple ex	High-dose (37) Low-dose (3)	Oncologic (37) Osteometabolic (3)	No antibiotic prophylaxis	Amox 500 mg TID × 14 d *If signs of infection at 14 d follow-up visit:* Amox 500 mg TID × additional 14 d (4 patients)	*Postop:* 0.12% CHX rinse BID until healing	Atraumatic ex, saline irrigation of socket and primary wound closure if possible	3 (2 wks; HD)	
Ristow et al., 2020 [37] Randomized pilot trial	*Subperiosteal flap group:* 82 (25-57)	68.4 ± 9.6 yrs	475 ex	High-dose (46) Low-dose (36)	Oncologic (46) Osteometabolic (36)	Amp-sulb 375 mg BID × 7 d *If allergy:* Clinda 600 mg × 7 d	Amp-sulb 375 mg BID × 7 d *If allergy:* Clinda 600 mg × 7 d	*Preop:* *0.2% CHX rinse tid × 2 d* *Postop:* *0.2% CHX rinse tid ≥ 5 d*	Sulcular incision, mucoperiosteal flap, alveoloplasty, primary wound closure Drug holiday: 30 d pre to 30 d postop after MD consult	5 (2 months; 3 HD, 2 LD)	
	*Epiperiosteal flap group:* 78 (18-60)	67.8 ± 10.1 yrs		High-dose (41) Low-dose (37)	Oncologic (41) Osteometabolic (37)				Sulcular incision, mucosal flap, alveoloplasty, primary wound closure Drug holiday: 30 d pre to 30 d postop after MD consult	18 (2 months; 13 HD, 5 LD)	
Miranda et al., 2021 [29] Retrospective study	*Study group:* 11 (0-11)	74.81 yrs	27 ex 18 ex mdb 9 ex max	High-dose (1) Low-dose (10)	Oncologic (6) Osteometabolic (5)	Amox-clav 1 g BID × 5 d + Metro 250 mg BID × 2 d *If allergy:* Azithro 500 mg 2h before ex	Amox-clav 1 g BID × 5 d + Metro 250 mg BID × 2 d *If allergy:* Azithro 500 mg OD × 5 d	N/A	Delicate curettage, PRF, suture without flap	0 (6 months)	
	*Control group:* 26 (1-25)	70.69 yrs	42 ex 27 ex mdb 15 ex max	High-dose (2) Low-dose (24)	Oncologic (12) Osteometabolic (14)				Delicate curettage, suture without flap	5 (6 months; 2 HD, 3 LD)	
Barry et al., 2021 [45] Retrospective study	652 (109-543)	69.7 yrs	652 ex 258 ex mdb 297 ex max 97 ex both	Low-dose (652)	Oncologic (9) Osteometabolic (643)	No antibiotic prophylaxis	*If high risk patients (low dose ≥ 4yrs or < 4yrs + corticosteroids)*: Amox 500 mg TID × 5 d *If allergy:* Metro 400 mg tid × 5 d	*Postop:* 0.2% CHX rinse tid until healing	Atraumatic ex	5 (12 months, LD)	
Pippi et al., 2021 [30] Retrospective case series	*Protocol n.2:* 13	68.02 ± 11.17 yrs	639 ex 321 ex mdb 318 ex max	High-dose (22) Low-dose (197)	Oncologic (22) Osteometabolic (197)	Amox 1 g BID × 3 d	Amox 1 g BID × 6 d	*Postop:* 0.2% CHX rinse × 7 d	Non-surgical ex, secondary intention healing	0(until mucosal healing, mean: 2 wks, range: 1–3 wks)	
	*Protocol n.3:* 206					Amox 1 g BID × 3 d + Metro 500 mg BID × 3 d	Amox 1 g BID × 6 d + Metro 500 mg BID × 6 d				
Parise et al., 2022 [43] Case–control study	*Group 1 (control):* 7 (2-5)	59.43 yrs	7 ex 5 ex mdb 2 ex max	High-dose (15)	Oncologic (15)	Amox 500 mg TID × 7 d + Metro 400 mg TID × 7 d	Amox 500 mg TID × 7 d + Metro 400 mg TID × 7 d	*Postop:* 0.2% CHX rinse BID	Mucoperiosteal flap (if necessary), bone edge smoothing, tension-free closure	2 (6 months; HD)	
	*Group 2 (prevention):* 8 (3-5)	58.38 yrs	8 ex 4 ex mdb 4 ex max						Mucoperiosteal flap (if necessary), bony edge smoothing, PRF, tension-free closure	0 (6 months)	
Seki et al., 2022 [41] Retrospective study	40 (N/A)	77.5 ± 7.9 yrs	70 ex 41 ex mdb 29 ex max	Low-dose (40)	Osteometabolic (40)	No antibiotic prophylaxis	Amox TID × 3 d	N/A	Drug holiday: 6.9 months (mean duration)	2 (122.6 ± 170 d; LD)	
Cuozzo et al., 2022 [31] Prospective study	*Low risk:* 20	67.5 ± 3 yrs	54 ex	Low-dose (45)	Osteometabolic (45)	Amox-clav 1 g BID × 3 d	Amox-clav 1 g BID × 3 d	*Preop:* 0.2% CHX rinse OD × 7 d *Postop:* 0.2% CHX rinse OD × 7 d	Atraumatic ex, bony edge smoothening, tension-free closure Drug holiday: 27 patients	0 (12 months)	
	*Low/medium risk:* 18		48 ex			Amox-clav 1 g BID × 3 d	Amox-clav 1 g BID × 12 d			1 (12 months; LD)	
	*Medium risk:* 3		38 ex			Amox-clav 1 g BID × 3 d	Amox-clav 1 g BID × 20 d			0 (12 months)	
	*High risk:* 4		19 ex			Ceftr 1 g IM × 2 d	Ceftr 1 g IM × 5 d + Amox-clav 1 g BID × 7 d			0 (12 months)	
Karaca et al., 2023 [50] Retrospective study	51 (14-37)	57.21 ± 10.21 yrs	109 ex 73 ex mdb 49 ex max	High-dose (51)	Oncologic (51)	Amox-clav 1 g BID × 2 d *If allergy:* Clinda 150 mg OD × 4 d	Amox-clav 1 g BID × 3 d *If allergy:* Clinda 150 mg OD × 3 d	*Postop:* 0.12% CHX rinse tid × 7 d	Atraumatic ex, osteoplasty, primary wound closure Drug holiday: 2 months (median duration), 31 patients	3 (8 wks; HD)	
Ristow et al., 2023 [38] Retrospective study	759 (219-540)	N/A	*Group 1 (IV):* 719 ex 368 ex mdb 351 ex max	High-dose (452) Low-dose (307)	Oncologic (452) Osteometabolic (307)	Amp-sulb 1.5 g × 1 d *If allergy:* Clinda 600 mg os or 600 mg/4 mL IV	Amp-sulb 1.5 g × 6 d If allergy: Clinda 600 mg os or 600 mg/4 mL IV	*Preop:* 0.2% CHX rinse TID × 10/14 d *Postop:* 0.2% CHX rinse TID × 10/14 d	Mucoperiosteal flap, alveoloplasty, primary wound closure Drug holiday: 30 d pre to 30 d postop after MD consult	50 sites	(3 months; 66 sites HD, 10 sites LD)
			*Group 2 (two wks):* 298 ex 150 ex mdb 148 ex max			Amox-clav 1 g × 7 d *If allergy:* Clinda 600 mg os or 600 mg/4 mL IV	Amox-clav 1 g × 7 d *If allergy:* Clinda 600 mg os or 600 mg/4 mL IV			17 sites	
			*Group 3 (one wk):* 126 ex 60 ex mdb 66 ex max			Amox-clav 1 g × 5 d *If allergy:* Clinda 600 mg os or 600 mg/4 mL IV	Amox—clav 1 g × 5 d *If allergy:* Clinda 600 mg os or 600 mg/4 mL IV			9 sites	
Megalhaes et al., 2023 [44] Case series	17 (2-15)	55 yrs	32 ex 10 ex mdb 22 ex max	High-dose (17)	Oncologic (17)	Amox 500 mg TID × 2 d *If allergy:* Clinda 600 mg TID × 2 d + Metro 400 mg TID × 2 d	Amox 500 mg TID × 8 d *If allergy:* Clinda 600 mg TID × 8 d + Metro 400 mg TID × 8 d	*Postop:* 0.12% CHX rinse TID until suture removal	Pentoxifylline 400 mg and tocopherol 400 IU tid 15 d preop and 15 d postop	3 (3 months; HD)	
Besi et al., 2024 [46] Observational study	*L-PRF group:* 15 (4-11)	75 yrs	22 ex 13 ex mdb 9 ex max	High-dose (2) Low-dose (13)	Oncologic (2) Osteometabolic (13)	No antibiotic prophylaxis	No postoperative therapy	*Postop:* 0.2% CHX rinse	Atraumatic ex, bony edge smoothening, PRF, primary closure (if possible)	0 (mean: 3 months)	
	*Control group:* 24 (6-18)	70 yrs	41 ex 20 ex mdb 21 ex max	High-dose (7) Low-dose (17)	Oncologic (7) Osteometabolic (17)				Atraumatic ex, bony edge smoothening, primary closure (if possible)	5 (mean: 10 wks; HD)	
Di Fede et al., 2024 [32] Randomized clinical trial	*Test group:* 38 (11-27)	66.7 yrs	N/A	High-dose (22) Low-dose (16)	Oncologic (26) Osteometabolic (12)	Amox-Clav 1 g TID × 1 d + Metro 500 mg TID × 1 d *If allergy:* Erythro 600 mg tid × 1 d + Metro 500 mg TID × 1 d	Amox-Clav 1 g TID × 6 d + Metro 500 mg TID × 6 d *If allergy:* Erythro 600 mg TID × 6 d + Metro 500 mg TID × 6 d	*Postop:* 0.2% CHX rinse	Alveoloplasty, O_2_-O_3_ injections (perialveolar and post-ex site), primary wound closure. Additional O_2_-O_3_ at 3–5 d, 14 d, and 6 wks	N/A	
	*Control group:* 79 (16-63)	69.6 yrs		High-dose (35) Low-dose (44)	Oncologic (37) Osteometabolic (42)				Alveoloplasty, primary wound closure		
Chang et al., 2025 [48] Retrospective study	329 (82-247)	74 yrs	836 yrs	High-dose (19) Low-dose (310)	Oncologic (19) Osteometabolic (310)	*Low risk:* No prophylaxis *If high risk:* Amox 2 g 1 h preop *If allergy:* Clinda 600 mg 1 h preop	*If high risk + immunocompromised, spreading odontogenic infection:* postoperative therapy	*Preop:* CHX rinse *Postop:* CHX rinse	Primary wound closure if tooth required to be surgically extracted	18 (8 wks; N/A)	

Legend: MRONJ, medication-related osteonecrosis of the jaw; N/A, not available; Preop, preoperative; Postop, postoperative; ex, extractions; mdb, mandibular; max, maxillary; Z, zoledronate; P, pamidronate; Amox-Clav, amoxicillin and clavulanic acid; Metro, metronidazole; Linco, lincomycin; Erythro, erythromycin; Clarithro, clarithromycin; Amp-Sulb, ampicillin and sulbactam; Penic, penicillin; IV, intravenous; Amox, amoxicillin; Amp, ampicillin; Clinda, clindamycin; Azithro, azithromycin; Ceftr, ceftriaxone; IM, intramuscular; h, hours; d, days; wks, weeks; yrs, years; BID, twice a day; TID, three times a day; OD, once a day; MD, medical doctor; CHX, chlorhexidine; PRF, platelet-rich factor; LLLT, low-level laser therapy; PBM, photobiomodulation; CTX, c-terminal cross-link telopeptide; aPDT, antimicrobial photodynamic therapy; FU, follow-up; BPs, bisphosphonates; HD, high-dose; LD, low-dose.

**Table 2:** Meta-analysis of MRONJ incidence in patients receiving high-dose versus low-dose therapy.

Analysis Model	OR	95% CI	z	*p*-Value
Common-effect model	3.26	1.74–6.10	3.69	0.0002
Random-effects model	2.82	1.46–5.43	3.08	0.0020

Legend: OR, Odds Ratio; 95% CI, 95% Confidence Interval.

**Table 3:** Final generalized linear mixed model for antibiotic duration and MRONJ risk (binomial, logit link; random intercept by study).

Predictor	β (Estimate)	SE	z Value	*p* Value	OR	95% CI (OR)
Intercept (low-dose, 0 days)	−5.75	0.76	−7.59	<0.001	0.003	0.001–0.012
Antibiotic days (low-dose)	+0.06	0.07	+0.93	0.352	1.06	0.93–1.22
High dose (vs. low dose)	+3.51	0.86	+4.10	<0.001	33.5	6.3–177.8
Days × High dose (interaction)	−0.15	0.07	−2.22	0.026	0.86	0.75–0.98

Legend: SE, Standard Error; OR, Odds Ratio; 95% CI, 95% Confidence Interval.

**Table 4:** Literature search strategy.

PubMed	Scopus	Web of Science
((“prevention” [All Fields] OR “antibiotic” [All Fields] OR “prophylaxis” [All Fields]) AND (“dental extraction” [All Fields] OR “extraction” [All Fields] OR “tooth extraction” [All Fields] OR “teeth extraction” [All Fields] OR “dental avulsion” [All Fields]) AND (“osteonecrosis” [All Fields] OR “osteonecrosis of the jaw” [All Fields] OR “bone necrosis” [All Fields] OR “jaw necrosis” [All Fields] OR “ONJ” [All Fields] OR “BRONJ” [All Fields] OR “MRONJ” [All Fields]) OR “ARONJ” [All Fields])	((“prevention” [all AND fields] OR “antibiotic” [all AND fields] OR “prophylaxis” [all AND fields]) AND (“dental extraction” [all AND fields] OR “extraction” [all AND fields] OR “tooth extraction” [all AND fields] OR “teeth extraction” [all AND fields] OR “dental avulsion” [all AND fields]) AND (“osteonecrosis” [all AND fields] OR “osteonecrosis of the jaw” [all AND fields] OR “bone necrosis” [all AND fields] OR “jaw necrosis” [all AND fields] OR “ONJ” [all AND fields] OR “BRONJ” [all AND fields] OR “MRONJ” [all AND fields]) OR “ARONJ” [all AND fields])	ALL = ((prevention OR antibiotic OR prophylaxis) AND (dental extraction OR extraction OR tooth extraction OR teeth extraction OR dental avulsion) AND (osteonecrosis OR osteonecrosis of the jaw OR bone necrosis OR jaw necrosis OR onj OR bronj OR mronj OR aronj))

## Data Availability

The data supporting this study’s findings are available from the corresponding author upon reasonable request.

## Abbreviations

The following abbreviations are used in this manuscript:

BID	twice a day
TID	3 times a day
MRONJ	Medication-related osteonecrosis of the jaw
HD	High-dose
LD	Low-dose
